# The Connection of the Genetic, Cultural and Geographic Landscapes of Transoxiana

**DOI:** 10.1038/s41598-017-03176-z

**Published:** 2017-06-08

**Authors:** Maxat Zhabagin, Elena Balanovska, Zhaxylyk Sabitov, Marina Kuznetsova, Anastasiya Agdzhoyan, Olga Balaganskaya, Marina Chukhryaeva, Nadezhda Markina, Alexey Romanov, Roza Skhalyakho, Valery Zaporozhchenko, Liudmila Saroyants, Dilbar Dalimova, Damir Davletchurin, Shahlo Turdikulova, Yuldash Yusupov, Inkar Tazhigulova, Ainur Akilzhanova, Chris Tyler-Smith, Oleg Balanovsky

**Affiliations:** 1 National Laboratory Astana, Nazarbayev University, Astana, Republic of Kazakhstan; 2 Vavilov Institute for General Genetics, Russian Academy of Sciences, Moscow, Russia; 3 Research Centre for Medical Genetics, Moscow, Russia; 4 L.N.Gumilyov Eurasian National University, Astana, Republic of Kazakhstan; 5 Leprosy Research Institute, Astrakhan, Russia; 6 Institute of Bioorganic Chemistry, Tashkent, Uzbekistan; 7 Center of High Technologies, Tashkent, Uzbekistan; 8 Institute of Strategic Research of the Republic of Bashkortostan, Ufa, Russia; 9 Forensic science centre of the Ministry of Justice of the Republic of Kazakhstan, Astana, Republic of Kazakhstan; 10 The Wellcome Trust Sanger Institute, Wellcome Genome Campus, Hinxton, United Kingdom

## Abstract

We have analyzed Y-chromosomal variation in populations from Transoxiana, a historical region covering the southwestern part of Central Asia. We studied 780 samples from 10 regional populations of Kazakhs, Uzbeks, Turkmens, Dungans, and Karakalpaks using 35 SNP and 17 STR markers. Analysis of haplogroup frequencies using multidimensional scaling and principal component plots, supported by an analysis of molecular variance, showed that the geographic landscape of Transoxiana, despite its distinctiveness and diversity (deserts, fertile river basins, foothills and plains) had no strong influence on the genetic landscape. The main factor structuring the gene pool was the mode of subsistence: settled agriculture or nomadic pastoralism. Investigation of STR-based clusters of haplotypes and their ages revealed that cultural and demic expansions of Transoxiana were not closely connected with each other. The Arab cultural expansion introduced Islam to the region but did not leave a significant mark on the pool of paternal lineages. The Mongol expansion, in contrast, had enormous demic success, but did not impact cultural elements like language and religion. The genealogy of Muslim missionaries within the settled agricultural communities of Transoxiana was based on spiritual succession passed from teacher to disciple. However, among Transoxianan nomads, spiritual and biological succession became merged.

## Introduction

**Figure 1:**
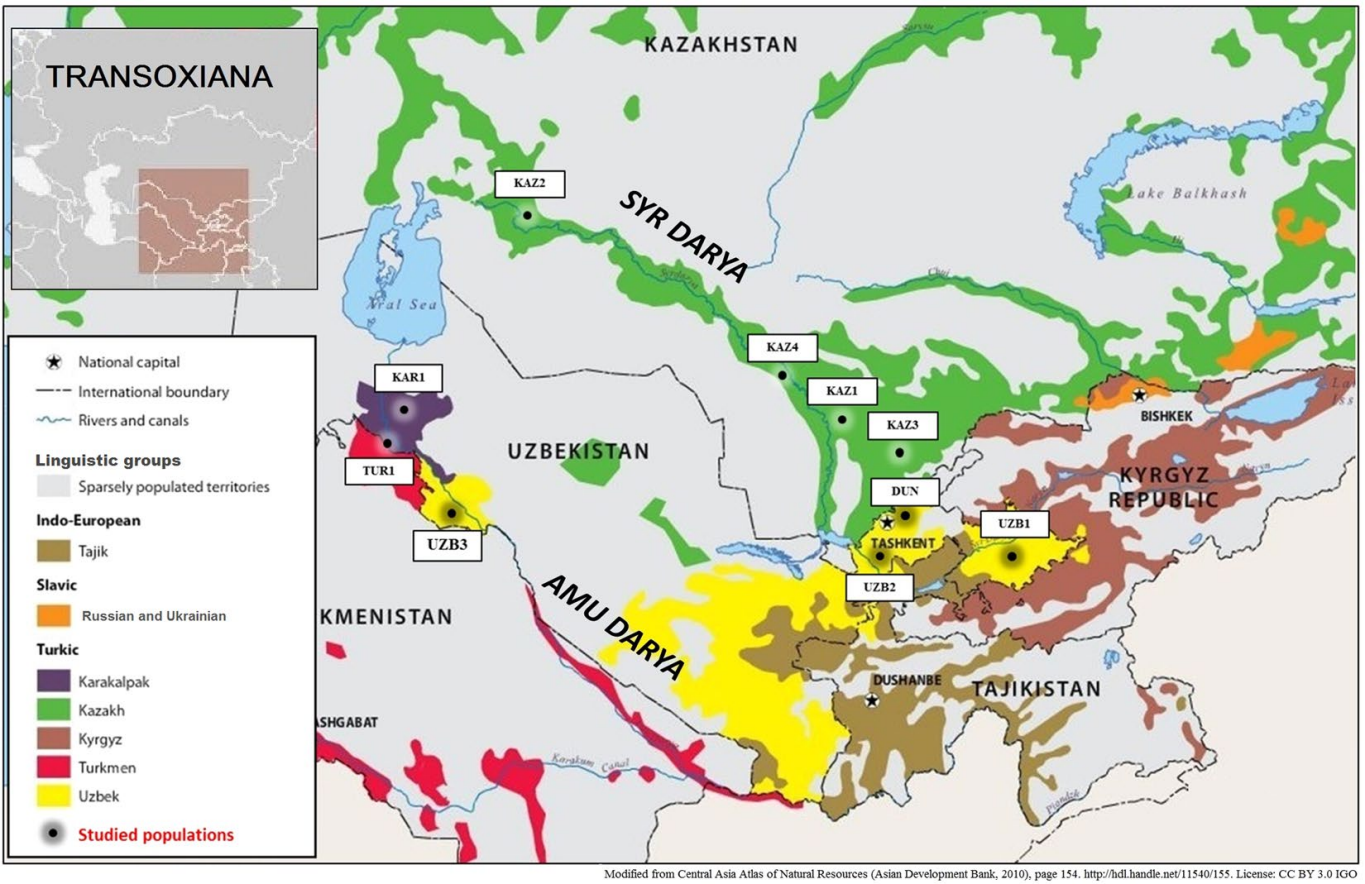
Map of Transoxiana and the populations studied. This figure is a derivative of Central Asia atlas of natural resources^76^ (http://hdl.handle.net/11540/155) by Asian Development Bank, used under CC BY 3.0 IGO. Areas with low population density (deserts and high mountains) are shown in grey.

Transoxiana is a historical region of Central Asia (Fig. 1). It covers the territories of five modern countries: Uzbekistan, western Tajikistan, western Kyrgyzstan, northwestern Turkmenistan and southern Kazakhstan. The peculiar features of its geographical landscape and abrupt shifts of cultural landscapes in the course of the history of the region allow us to use it as a model for investigating the connection between genetic, cultural and geographic landscapes.

*Geographic landscape of Transoxiana*. This includes a desert located between the Amu Darya and Syr Darya river basins. The riversides are densely populated by Kazakhs, Uzbeks, Karakalpaks, Kyrgyz and Turkmens (Fig. 1). The Tian Shan mountains are located in the southeast of Transoxiana, while desert plains expand to the northwest and are bordered by the Aral Sea. As geographical landscapes and barriers tend to shape the gene pool^1–3^, it is important to assess their role in the geographically heterogeneous Transoxiana.

*Cultural landscape of Transoxiana*. Two distinct modes of subsistence with contrasting traditional cultures - settled agriculture and nomadic pastoralism - have been practiced in the region for thousands of years. Located on the Silk Road, Transoxiana’s history has been influenced by both the West and the East. The first known impact came from Western Asia. During the rule of the Achaemenid Empire (6th century BC) the region was at the center of the Sogdian culture. In the 4th century BC Alexander the Great turned it into a Hellenistic province, naming it Transoxiana (“area beyond the Ox river”; Ox is the ancient name for Amu Darya). Later the region was part of the Seleucid Empire (4th century BC), the Greco-Bactrian Kingdom (3rd - 2nd centuries BC), the Kushan Empire (1st century BC – 5th century AD), the Hephthalite Empire (5th century AD), and the Sasanian Empire (6th century AD). In the 5th century AD the direction of migration changed to Central Asian sources: with the expansion of Turkic tribes, the region becomes part of the Turkic Khaganate (7th century AD) and succeeding realms. The source of migration returned to Western Asia in the 8th century AD: due to the expansion of Islam, the region fell under the influence of the Arab culture and became known as Mawarannahr. In the 13th century AD the migration vector switched again to a Central Asian source: most of the region was under Mongol cultural influences, becoming the Chagatai Khanate - part of the Mongol Empire^4–6^. Besides these major cultural influences, minor ones such as a small settlement of Han Chinese mentioned by the Buddhist monk Xuanzang in 630 BC took place^4^.

Thus the major landmarks in Transoxiana’s history were: the development of settled agriculture and nomadic pastoralism, the influence of Western Asian Empires, Turkic nomads, Arabs and Mongols. The expansion of Islam by Arabs and preservation of both types of economy (settled agriculture and nomadic pastoralism) had the biggest impact on the cultural landscape. Though cultural expansion itself is of course nothing more than changes of cultural elements (language, religion, technology), it is often associated with demic expansion (human migration) that has direct influence on the gene pool. A number of studies have exemplified influences of religious^7, 8^ or linguistic shifts^9–11^ on genetic structure.

*Genetic landscape of Transoxiana*. Previous genetic studies in Central Asia have used autosomal^12, 13^, mtDNA^14–16^ or Y-chromosomal markers^17–24^. All these studies stressed the pronounced heterogeneity of the genetic landscape in Central Asia. Most recent studies were dedicated to Y-chromosomal variation, in particular regions or clans within Central Asia^25–33^.

In line with these studies, we also concentrate here on a single region within Central Asia. The Y-chromosomal variation in this Transoxiana region has been insufficiently studied so far. 133 samples from Kyrgyz and Kazakh populations were studied using 8 Y-STRs^17^. 150 samples from Kazakh, Kyrgyz, Uzbeks, Turkmens, and Tajiks were studied using 16 STRs and 16 SNPs^20^. Subsequent studies^22–24^ were updated recently^30^, presenting variation of 8 Y-STRs and 31 SNPs in 461 samples. Here, we double this number by genotyping 17-STRs and 35 SNPs in 780 samples, including four regional Kazakh populations along the Syrdarya river, three regional Uzbek populations, Karakalpaks, Turkmens and Dungans.

The key element of Transoxiana populations that influences both genetic and cultural landscapes is the tribal-clan structure prevalent among nomadic populations. Many modern Kazakh and Turkmen individuals identify themselves not only with an ethnic group, but with a clan as well. Genealogical lineages are combined into clans, and clans are combined into tribes. The tribal-clan structure is based on the traditional belief in the unified bloodline of an ethnic group and has long regulated the ethnosocial order of nomadic populations. This tradition has endured through all the cultural expansions and has incorporated the new elements into the tribal-clan structure. Often the name of the lineage, clan and tribe are inherited through the male line just as the Y-chromosome is. Therefore, it is important to study them together^22, 33^. The areas studied here are predominantly populated by the Kazakh tribe Konyrat, the Kazakh clans Alimuly, Kozha (Khoja) and Sunak, as well as the Turkmen tribe Yomut. A brief summary of the genealogy and history of these clans is given in the Supplementary Text.

This study aims to examine the genetic landscape of Transoxiana and explore its connection to geographical and cultural landscapes. To achieve this aim, we examined Y-chromosomal variation in Kazakhs, Uzbeks, Karakalpaks, Turkmens and Dungans with reference to their cultural and geographical landscapes. The results were used to determine whether or not the two last major expansions (Arab and Mongol) were demic and influenced the Y-chromosomal variation in Transoxiana.

## Results and Discussion

### Genetic portraits of populations and tribal/clan groups of Transoxiana

**Figure 2:**
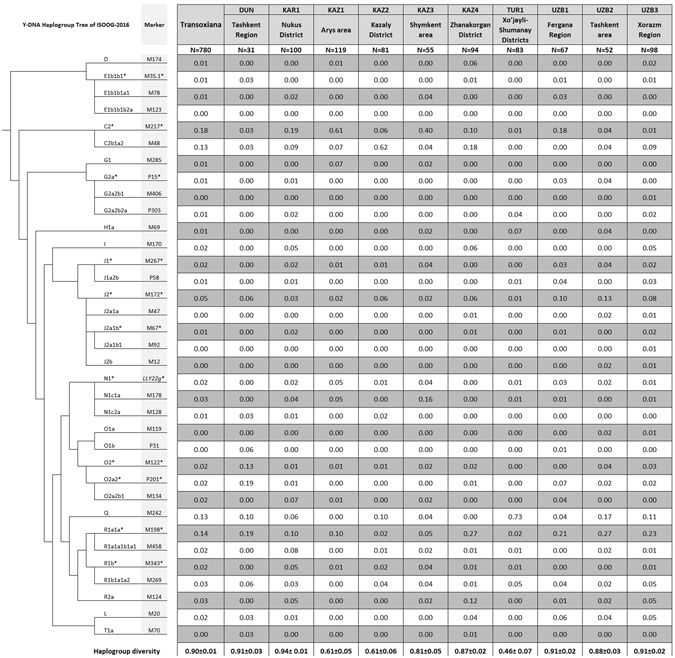
Frequencies of Y-chromosomal haplogroups in Transoxiana populations.

780 samples from 10 populations - Kazakhs (4 regional populations, coded from KAZ1 to KAZ4, see Fig. 2 and Supplementary Table 2), Uzbeks (3 regional populations, UZB1 to UZB3), Karakalpaks (KAR1), Turkmens (TUR1) and Dungans (DUN) - were studied using 35 binary and 17 STR Y-chromosomal markers (Figs 1 and 2, Supplementary Table 1). Data on 5,218 samples from 69 populations, representing 16 Asian countries ranging from Turkey to China (Supplementary Table 2), were used to provide a genetic context. More than half of the Transoxianan Y-chromosomes (58%) falls into three haplogroups (Fig. 2): C2-М217 (31%), R1a1a-M198 (16%), and Q-M242 (13%), although the distribution of these haplogroups in Transoxiana is uneven. C2-М217 comprises almost two-thirds of the south Kazakh gene pool (61% C2*-М217(хМ48) among KAZ1, 62% C2b1a2-М48 among KAZ2). R1a1a-M198 is common among Uzbek (UZB1, UZB2, UZB3, 21-29%), in one Kazakh population (KAZ4, 28%) and among Dungans (DUN, 19%). Q-M242 is found in more than two-thirds of the Turkmen sample (TUR1, 73%).

The prevalence of specific haplogroups is even more pronounced for tribal-clan groups than for geographic populations (Supplementary Fig. 1): C2*-М217(хМ48) comprises 88% of the Y-chromosomes of the Konyrat tribe, C2b1a2-М48 reaches 75% in the Kazakh clan Alimuly, and Q-M242 accounts for 71% in the Turkmen tribe Yomut. Based on haplogroup frequency, the Konyrat tribe is the most homogenous (HD = 0.23), while the Kozha-Sunak clan group is the most heterogeneous (HD = 0.94). The specificities of the clan pools of paternal lineages are the reason for the specificities of the geographic populations: the clan Alimuly prevails in the KAZ2 population (79% samples are from this clan), the tribe Konyrat predominates in the KAZ1 population (62%), and the tribe Yomut predominates in the TUR1 population (88%).

### The Transoxianan paternal heritage in the Asian context

**Figure 3:**
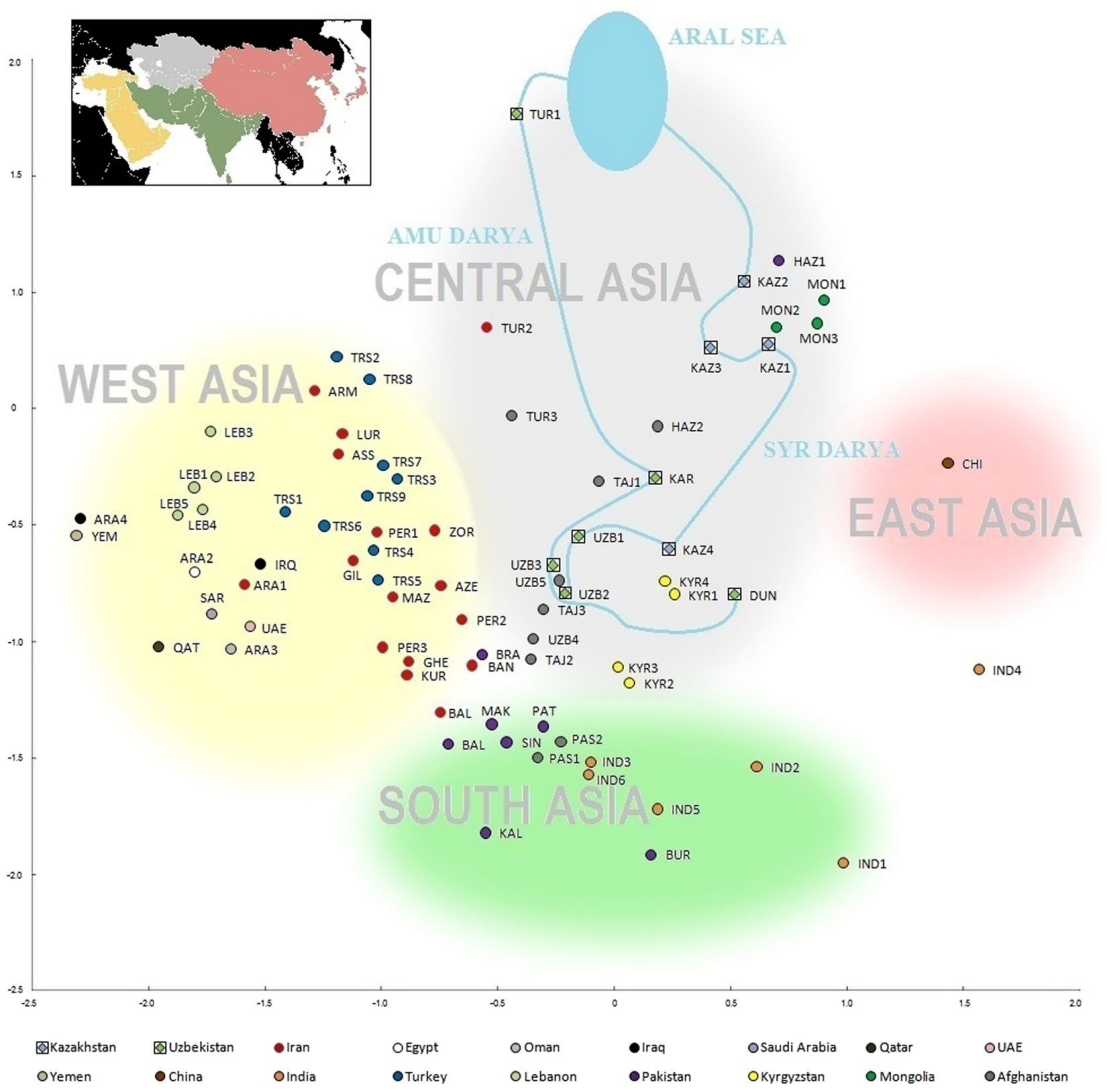
Genetic relationships of Asian (including Transoxiana) populations using 30 Y-SNPs. Multidimensional scaling plot; stress = 0.17. Populations from 18 countries are marked by colors. The ten populations from this study are shown as rhombuses within squares, while populations from the literature are indicated by circles. Blue lines link populations located along the Amu Darya and Syr Darya rivers. Population codes are explained more fully in Supplementary Table 2. Colored cloud areas represent geographic clusters, with colors on the main plot following colors on the inset (Asian regions according to UN classification).

The 10 populations from Transoxiana were analyzed along with 69 other Asian populations typed by the same panel of 30 Y-chromosomal SNPs (Supplementary Tables 2 and 3). Clusters corresponding to geographic parts of Asia were revealed in the multidimensional scaling plot (Fig. 3). The Western Asian cluster was represented by Arab, Turkish and Iranian populations. Populations of India, Pakistan and Afghanistan made up Southern Asian cluster. Chinese form the Eastern Asia cluster. All Transoxianan populations lie in the Central Asian cluster.

Analysis on a narrower geographic scale (Transoxiana and the neighboring regions) is available in Supplementary Fig. 3 (Supplementary Table 4). This PCA plot is based on a smaller number of haplogroups, but includes more Central Asian populations. Both an MDS plot of 30 haplogroups and a PC plot of 19 haplogroups (Fig. 3, Supplementary Fig. 2, Supplementary Fig. 3) demonstrate the four following patterns.

First, Uzbek and Tajik populations practicing settled agriculture, as well as Kyrgyz, are genetically distant from most nomadic populations (Mongol, Kazakh, Hazaras). Second, despite originating from three countries (Uzbekistan, Iran, Afghanistan), Turkmen populations form their own firmly separated cluster. The reason lies in high frequency of haplogroup Q-M242 (Supplementary Table 2) in most Turkmen populations, which in particular forms the third PC (Supplementary Fig. 2), though this haplogroup is absent from the fourth Turkmen population^30^ despite their sample coming from the same region of Uzbekistan as our sample. Third, Dungan populations of Uzbekistan (DUN) are genetically closer to the populations of China (genetic distance d = 0.178) and northeastern India (d = 0.152) than to their neighboring Uzbek populations (d = 0.228; d = 0.410; d = 0.425). This is explained by the historically recent migration of Dungans from China and the maintenance of their Sino-Tibetan language, prevalent in China and northeastern India. Fourth, most of the Kazakh populations studied cluster with Mongols, Pakistani Hazaras (HAZ1) and Afghan Hazaras (HAZ2) due to their high frequency of haplogroup C2-М217. This correlates with the historically well-known Mongol origin of the Hazaras^34, 35^. In addition, Fig. 3 shows the populations located along a stretch of the Amu Darya and Syr Darya rivers linked by blue lines symbolizing the rivers. However, the positions of these genetic “rivers” only loosely correlate with their geographical prototypes.

### The lack of relationship between genetics and geography

To determine the driving forces that shaped the Y-chromosomal variation in Transoxiana, we examined patterns of genetic variation by AMOVA (Supplementary Table 5). Populations were arranged into groups in three ways: (a) Geography – river basins: populations from the Amu Darya or Syr Darya basins; (b) Geography - altitude: plain or foothill populations (400 meters altitude was used as a threshold); (c) Mode of subsistence: settled agriculturalists or nomadic pastoralists.

Both ways of geographic grouping had little to no influence on the genetic structure (Table 1). A Mantel test (Yr = −0.006, p = 0.44) further supports the idea that, unlike most other regions, in Transoxiana genetic distances between populations do not correlate with the geographic distances. But the mode of subsistence had a significant impact on explaining the genetic structure: the differences between settled and nomadic populations accounts for 2.85% of the total genetic variation, which is almost three times larger than the differences between the geographic groups of populations (Table 1).

**Table 1:** Variation in Y-Chromosomal haplogroup frequencies between groups.

Basis of Division	Groups	Percentage of Variation		
		Within populations	Among populations within groups	Among groups
Geography 1	Amu-Darya Syr-Darya	84.18*	16.04*	−0.22 (p-value = 0.39)
Geography 2	Foothills Plain	82.43*	16.52*	1.05 (p-value = 0.27)
Mode of Subsistence	Settled agriculture Nomadic pastoralism	82.82*	14.33*	2.85 (p-value = 0.096)

*p-value < 0.01.

From the 5^th^ to 2^nd^ millennia BC a complete transition to a cattle-raising and agricultural tribal existence occurred in Transoxiana populations^36^. Since that time, the mode of subsistence - settled agriculture or nomadic pastoralism - was the main cultural distinction within Central Asia. This lets us conclude that the influence of geography on the genetic structure was mediated by a combination of subsistence and traditional culture. One may suppose that such relationships of cultural and geographical factors have persisted for thousands of years. It underlines the important role which technical innovations and culture often play in shaping the genetic landscape^37^.

### Arab and Mongol expansions: migration of cultures or populations?

In order to search for signs of male demic expansions, we identified four modal STR-haplotypes of Transoxiana (those present in more than 10 samples in our dataset, Table 2). For each modal haplotype we then identified related haplotypes. We considered haplotypes which were fewer than 5 mutational steps from the modal haplotype and belong to the same haplogroup. Five mutations - considering 15 Y-STRs and mutation rate 0.0021 per locus per generation - might occur within roughly two thousand years, which covers the time interval important for our analysis. The search for related haplotypes was performed in a database of 4495 Y-STR Asian haplotypes using the Haplomatch software^38^. This methodology is similar to that applied by Balaresque and colleagues^30^ in their search for Asian primary descent clusters.

**Table 2:** Features of the primary descent clusters.

Modal haplotype	N*	N**	Haplotype cluster	N***	SNP-marker	DYS389I	DYS389b	DYS390	DYS456	DYS19	DYS19-2****	DYS385a	DYS385b	DYS458	DYS437	DYS438	DYS448	GATA_H4	DYS391	DYS392	DYS393	DYS439	DYS635	TMRCA (years) of cluster	
																								Rho	ASD
Modal haplotype1	26	257	α	67	M48	14	17	25	15	16	—	12	12	17	14	10	20	10	10	11	13	11	23	600 ± 200	580
			β	188	M48	14	17	24	15	16	17	12	12	18	14	10	20	10	9	11	13	11	23	800 ± 200	659
Modal haplotype2	15	138	σ	76	M217(xM48)	14	15	23	15	15	—	11	20	18	14	10	21	11	10	11	14	12	21	1100 ± 400	1161
			γ	53	M217(xM48)	13	15	23	15	15	—	11	18	18	14	10	21	11	10	11	14	11	22	600 ± 200	704
Modal haplotype3	12	189	μ	185	M217(xM48)	13	16	25	15	16	—	12	13	18	14	10	22	11	10	11	13	10	21	1100 ± 300	1298
			λ	19	M217(xM48)	13	16	25	15	16	—	12	13	17	14	10	22	12	10	11	13	10	21	400 ± 100	407
Modal haplotype4	11	98	δ	97	M242	13	15	23	17	13	—	13	16	19	14	11	22	11	10	16	13	13	23	1400 ± 500	1360

Notes: *Number of samples carrying the modal haplotype;

**number of samples carrying related haplotypes (fewer than 5 mutational steps from the modal haplotype);

***number of samples within the given cluster.

****Duplication of the DYS19 locus was observed only in some of M48 haplotypes.

Each ASD estimate falls within confidence interval of the corresponding rho estimate, so we mention mainly rho estimates in the text.

The modal haplotype 1 (Table 2) and 257 related haplotypes belonging to haplogroup C2b1a2-M48 were used to construct a phylogenetic network (Supplementary Fig. 4A). Two clusters can be distinguished: cluster α (which includes the modal haplotype) and cluster β. Cluster α is 600 ± 200 years old and its modal haplotype is most prominent among the Kazakh clan Alimuly (33%). Cluster β is mostly present among Mongols and Mongolian-speaking Kalmyks. Cluster β is older (800 ± 200 years using the rho estimate and 660 years using ASD, Table 2), suggesting the gene flow took place from Mongolia to Transoxiana rather than in the reverse direction. The age of the cluster overlaps with the formation of the Mongol Empire (13th century AD) making this suggestion plausible.

The modal haplotype 2 (Table 2) and 138 related haplotypes belonging to the C2*-M217(xM48) haplogroup were arranged into a second phylogenetic network (Supplementary Fig. 4B). Here as well, two clusters can be distinguished: γ and σ. Cluster γ is prevalent among Mongolian-speaking Kalmyks and in Mongolia itself. The age of this cluster (600 ± 200 years) overlaps with the time of migration of the Kalmyk ancestors (Oyrats) from Mongolia and the following back migration of some Kalmyk groups. Cluster σ is specific to the Kazakh tribe Konyrat and modal haplotype accounts for 17% of the tribal paternal pool. The age of this cluster (1100 ± 400 years old) suggests a fairly early migration from Mongolia followed by an expansion within the single tribe.

The modal haplotype 3 (Table 2) and 189 related haplotypes belonging to the C2*-M217(xM48) haplogroup were plotted similarly (Supplementary Fig. 4C). This haplotype coincides with a previously-described haplotype, putatively connected to Genghis Khan’s relatives, collectively forming the “С3* star-cluster” (μ)^21^. From Abilev *et al*.^26^ it is known that 76.5% of the Kazakh tribe Kerey belong to the star-cluster, including the 16% that fall within the third modal haplotype in our classification. Within Transoxiana, this founder haplotype is most common among the Kazakh clan Tore (11%), tribe Uysun (6%) and Karakalpaks (5%). The estimated age of the μ cluster (1100 ± 300 years) aligns with previous estimations of ~1000 years^21, 30, 39^. It may be assumed that modal haplotype 3 was the “proto-Mongolian haplotype”, inherited, among others, by Genghis Khan, his descendants and patrilineal relatives. It is important to mention that Temujin (Genghis Khan) belonged to the Kiyat clan, which in turn is a branch of the Borjigin tribe, part of the Nirun Mongols. Subcluster λ, aged 400 ± 100 years old, is specific for Hazara from various countries and can be distinguished within the cluster.

The modal haplotype 4 (Table 2) and 97 related haplotypes belonging to the Q-M242 haplogroup were again plotted on a network (Supplementary Fig. 4D). The overwhelming majority of these haplotypes came from Turkmen populations from several countries. The cluster δ is 1400 ± 500 years old, making it older than the Mongol expansion. Despite a small part of the confidence interval overlapping with the period of Arab expansion, haplogroup Q-M242 accounts for just 1.5% of the population of the Arabian Peninsula, which means that expansion of this cluster in Turkmen populations is more likely caused by a local founder effect predating both Arab and Mongol influences.

Thus, three out of four signals of expansion in Transoxiana are connected to Mongol populations and likely reflect the migration to Transoxiana from Mongolia or neighboring regions which was followed by rapid growth of the migrants’ descendants (Supplementary Text). Notably, such successful demic expansion was not accompanied by cultural expansion (language change) - most populations of present-day Transoxiana speak not Mongolian, but Turkic languages. The factor that unifies not just most, but all, populations of Transoxiana is Islam. However, our analysis has not revealed any signs of significant demic expansions linked to the Arabs. In a more direct attempt to uncover signs of such expansions, we have analyzed the Y-chromosomes of nomadic Islamic clergy.

### In search of Arab ancestry in Transoxiana

The spread of Islam was one of the most powerful cultural expansions of West, South and Central Asia. There are ethnographic and genealogical findings identifying the demic traces of Arabs in Transoxiana. In particular, the clans Kozha and Sunak are traditionally considered as descendants of the Prophet Muhammad’s close paternal-line relatives (Supplementary Text). These clans have maintained a privileged position among the nomadic populations of Transoxiana with a status similar to Genghis Khan’s relatives, who were treated as nobility. While our previous study of the Kozha clan^39^ was based on haplogroup frequencies, in this study we have analyzed STR haplotypes of the Kozha and Sunak clans.

**Figure 4:**
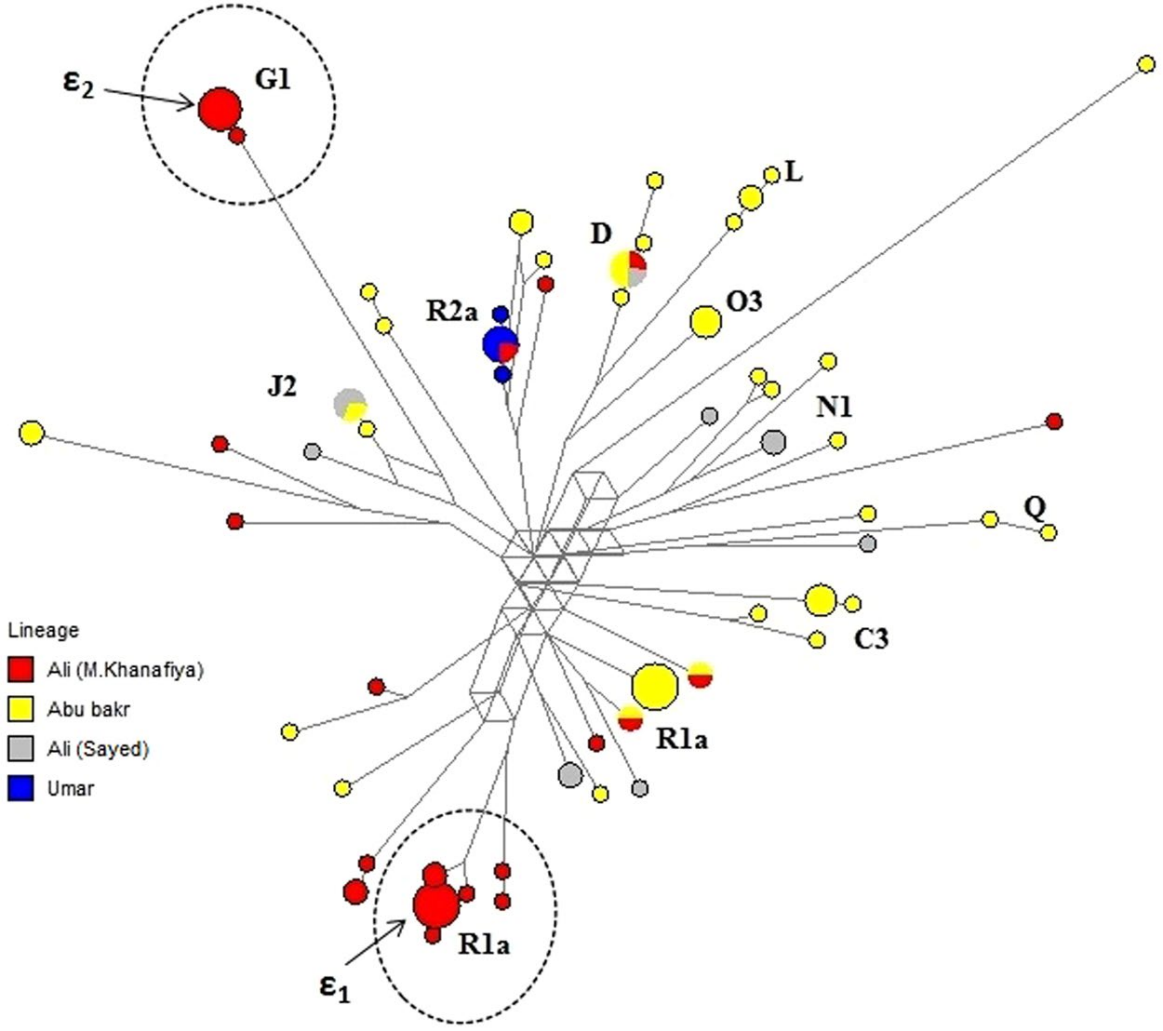
Haplotypic diversity of genealogical lineages within the Kozha-Sunak tribal-clan group, represented on a median-joining network.

Y-chromosomal haplotypes of the Kozha-Sunak tribe are shown in Fig. 4. Many separate individual haplotypes, or sometimes mini-clusters, can be observed. Therefore, unlike most Transoxianan clans, the Kozha and Sunak clans do not have a predominant paternal common ancestor. This is confirmed by the high haplogroup variation (HD = 0.86) among the Kozha-Sunak, which is 2–4 times higher than all other Transoxianan lineages studied (Supplementary Fig. 2). Due to the fact that subclans can have different origins, we divided the Kozha-Sunak into four groups based on traditional genealogy, and likewise divided all other Transoxianan clans. In the course of this analysis (Supplementary Table 6) we have discovered a pattern: the Kozha-Sunak lineages are highly heterogeneous based on the mean number of pairwise differences between haplotypes (PD between 8 and 9), while subclans of other Transoxianan clans are relatively homogeneous (PD between 1 and 7). The absence of one principal male root in the Kozha-Sunak tribe is indicative of its origin not being one Arab ancestor. Furthermore, haplogroup J1-M267, considered a marker of Arab expansion^40^, was not found among the Kozha-Sunak.

In order to trace the origin of the haplotype mini-clusters identified (Fig. 4), we searched for related haplotypes (fewer than 5 mutational steps) in other Asian populations. Mini-cluster R1a (ε1) had no related haplotypes, but mini-cluster G1 (ε2) has related haplotypes among the Kazakh tribe Argyn^33^. This suggests that the origin of this mini-cluster is local and has not migrated with the Arab expansion.

Sayeds (lineage within the Kozha and Sunak clans) are known as descendants of the Prophet Muhammad on the paternal side, and reside beyond Transoxiana as well. Sayeds of Pakistan, for example, were analyzed by Belle *et al*.^41^, who reported that they are genetically closer to Arabs than to the surrounding populations of Pakistan and India, but did not find a founder effect. Furthermore, the Pakistani Sayeds are quite different genetically from the Transoxianan Sayeds. Thus, despite traditionally attributing their paternal ancestry to one common root among Arab missionaries, the pronounced Y-chromosomal variation suggests that Transoxiana’s lineages have descended from several unrelated local ancestors.

A possible explanation is that nomadic clergy genealogy was based on *silsila*, a spiritual legacy passed from teacher to disciple, rather than a biological relationship. In Central Asia, Islam was spread by the Sufi orders Yasawiyya, Naqshbandi and Bektashi. In these orders, the leadership was based on *silsila*, a sequence of teachers who taught the succeeding leader of the Sufi order. However, even in agriculturalists spiritual succession was sometimes passed from father to son^42^ and when occurring within a nomadic patronymic tradition could become a patrilineal biological legacy. The age of mini-cluster ε1 (600 ± 200 years old) corresponds to the time when the Golden Horde adopted Islam as the official religion, and the rise of the Kozha-Sunak tribal-clan group in social status. This may have facilitated the transition from spiritual *silsila* to biological genealogy in order to maintain the privileged social status within the tribal-clan group. This conclusion coincides with the supposition of Heyer *et al*.^43^ that cultural transmission of reproductive success could play an important role in shaping genetic diversity in Central Asia.

## Conclusions

We have analyzed human Y-chromosomal variation in ten populations from Transoxiana, a historical region covering Uzbekistan, western Tajikistan, western Kyrgyzstan, northwestern Turkmenistan and southern Kazakhstan. Considering the peculiar features of the geographical landscape of the region, abrupt shifts of cultural landscapes in the course of its history, and presence of patrilineal tribal-clan groups, we jointly analyzed the patrilineal genetic variation, patrilineal genealogies and historical data. We identified three features of the genetic landscape of Transoxiana and its connection to geographical and cultural landscapes.

First, cultural and demic expansions of Transoxiana were not closely connected with each other. Arab cultural expansion introduced Islam to the region but did not leave a significant mark on the Y-chromosomal pool. The Mongol expansion, in contrast, had enormous demic success, but did not impact on cultural elements like language and religion.

Second, the geographic landscape of Transoxiana, despite its peculiarity and diversity (deserts, fertile river basins, foothills and plains) had no strong influence on the genetic landscape. The main factor structuring the Y-chromosomal variation was the mode of subsistence: settled agriculture or nomadic pastoralism.

Third, the genealogy of Muslim missionaries within the settled agricultural community was based on spiritual succession passed from teacher to disciple, rather than on biological relationship. However, among nomads, spiritual and biological succession merged, leading to the formation of haplotype mini-clusters among nomadic clergy.

## Methods

### Samples

Blood samples were collected in 2009–2012 by the expeditions of the Laboratory of Human Population Genetics of the Research Centre for Medical Genetics, Genome Geography Laboratory of the Vavilov Institute of General Genetics, The National Laboratory Astana of Nazarbayev University, Forensic science centre of the Ministry of Justice of the Republic of Kazakhstan (Astana), Center of High Technologies and Institute of Bioorganic Chemistry (Tashkent), partially under the auspices of the Genographic Project. All expeditions were supervised by Elena Balanovska and followed the same sampling rules^44^.

10 populations of 5 ethnic groups were studied (Supplementary Table 1). Only individuals who had all their ancestors for at least three generations descending from the specific population, and not related to each other, were selected. All 780 subjects provided their written informed consent in a form approved by the Ethics Committee of the Research Centre for Medical Genetics (Moscow, Russia). When performing genotyping and data analyses, we followed lab protocols approved by the same Ethics Committee.

The MDS analysis dataset consisted of 5,998 samples from 79 populations, including 780 samples reported here for the first time (Supplementary Table 2)^7, 29, 45–52^. PCA included data on 1,944 samples from 33 Central Asian populations (Supplementary Table 4)^29, 30, 50^ and this study.

The phylogenetic analysis dataset included 15-STR profiles of 4,495 samples from Central Asia and neighboring regions^2, 25–29, 33, 45, 48, 53–55^ and this study (Supplementary Fig. 4A–D). Both the MDS and the phylogenetic dataset were extracted from the in-house *Y-base* database, compiling published data on Y-chromosomal variation in human populations.

### Genotyping

DNA was extracted from white blood cells of peripheral blood using standard methods^56^. The effective DNA concentration was determined by real-time PCR using the Quantifiler Human DNA Kit (Applied Biosystems), followed by normalization of DNA to a concentration of 2 ng/µl.

To determine the Y-chromosome haplogroup, 35 SNP markers were genotyped (M217, M48, M174, M35.1, M78, M123, M285, P15, P303, M406, M69, M170, M267, P58, M172, M47, M67, M12, M92, M20, LLY22g, M128, M178, M119, P31, M122, P201, M134, M242, M198, M458, M343, M269, M124, M70) using the TaqMan probes on the 7900HT instrument (Applied Biosystems) according to the manufacturer’s protocol. Haplogroups were classified according to ISOGG-2016 (Version: 11.208; Date: 30 July 2016)^57^ (Fig. 2, Supplementary Fig. 1, Supplementary Table 1).

17 Y-STR loci were genotyped in all samples (DYS389I, DYS389II, DYS390, DYS456, DYS19, DYS385a, DYS385b, DYS458, DYS437, DYS438, DYS448, GATA_H4, DYS391, DYS392, DYS393, DYS439, DYS635) using the Yfiler PCR Amplification Kit (Applied Biosystems) on the ABI 3130xl genetic analyzer (Applied Biosystems). The results were analyzed using the GeneMapper Software v. 4.1 (Applied Biosystems).

### Statistical methods

Genetic distances between 79 populations were calculated using Nei’s method and DJ software (Supplementary Table 3)^58^. Multidimensional scaling, cluster analysis (Ward’s method), and principal component analysis were conducted using the Statistica v.7.1 software^59^. Genetic differentiation within and among groups of populations (AMOVA) was performed in Arlequin 3.5.1.3 software^60^.

15-STR-haplotypes were analyzed (DYS385a and DYS385b were excluded; for DYS389, DYS389I and DYS389b [=DYS398II-DYS389I] were used). Haplotype variability statistics were calculated using the Arlequin 3.5.1.3 software. The search for related haplotypes was conducted using the Haplomatch software^38^. Phylogenetic analysis was conducted using the Reduced-Median method^61^ in Network 5 software^62^ (http://www.fluxus-engineering.com/sharenet.htm) with reduction limit = 1. The resulting phylogenetic networks were then edited using Network Publisher^63^ (http://www.fluxus-engineering.com/nwpub.htm).

In distinguishing the clusters on the networks, we followed the procedure described earlier^11, 64^. Briefly, we first looked for a zone in the network carrying mostly haplotypes from a single population; then the most recent common ancestral haplotype for all haplotypes in the zone was identified in the network; finally, all haplotypes downstream to this ancestral haplotype were attributed to the cluster, though some very distant haplotypes were ignored. This procedure transforms the initial arbitrary “zone” to a monophyletic – to the best of the network’s performance – clade. In previous studies^64^ we selected the ancestral haplotype as cluster’s founder, but^65^ found that using the modal haplotype works much better, so here we selected modal haplotypes as founders. Cluster age was determined using the rho-statistic^66, 67^ and, because rho was shown to introduce a systematic bias^68^, we also used the average squared distance (ASD) estimator^69, 70^. Rho was calculated using Network 5, and ASD was calculated by Y TMRCA Calculator (http://ehelix.pythonanywhere.com/init/default/about), which is a derivative of Matlab/Octave program Ytime^71^. To convert the number of mutations into number of generations, the “genealogical” mutation rate of 2.1 × 10^−3^ mutations per STR per generation was used^72, 73^ as the analysis in Karmin^74^ indicated that for clusters younger than 30,000 years, this rate is consistent with full Y-chromosomal sequence data. When converting the number of generations into an age in years, the male generation time was set to 30 years^75^. To determine whether or not most haplotypes in the same genealogical lineage originated from a common ancestor, we used phylogenetic networks, haplotype variability statistics and the mean number of pairwise differences within each lineage (calculated using Arlequin 3.5.1.3 software).

## Electronic supplementary material

Supplementary Dataset

Supplementary information
