# Supplementary material for: The Connection of the Genetic, Cultural and Geographic Landscapes of Transoxiana
Source: Sci Rep. 2017 Jun 8;7:3085. doi: 10.1038/s41598-017-03176-z (PMC5465200; doi:10.1038/s41598-017-03176-z)
Supplement: Supplementary file 2 — Supplementary information [file 41598_2017_3176_MOESM2_ESM.pdf]

Supplementary information

The Connection of the Genetic, Cultural and Geographic Landscapes of Transoxiana

Maxat Zhabagin[1,2]*¶, Elena Balanovska[3]¶, Zhaxylyk Sabitov[4], Marina Kuznetsova[3], Anastasiya Agdzhoyan[2,3], Olga Balaganskaya[2], Marina Chukhryaeva[2,3], Nadezhda Markina[2], Alexey Romanov[3], Roza Skhalyakho[2,3], Valery Zaporozhchenko[2,3], Liudmila Saroyants[5], Dilbar Dalimova[6], Damir Davletchurin[6], Shahlo Turdikulova[7], Yuldash Yusupov[8], Inkar Tazhigulova[9], Ainur Akilzhanova[1], Chris Tyler Smith[10], Oleg Balanovsky[3]*

[1]National Laboratory Astana, Nazarbayev University, Astana, Republic of Kazakhstan
[2]Vavilov Institute for General Genetics, Russian Academy of Sciences, Moscow, Russia
[3]Research Centre for Medical Genetics, Russian Academy of Medical Sciences, Moscow, Russia
[4]L.N.Gumilyov Eurasian National University, Astana, Republic of Kazakhstan
[5]Leprosy Research Institute, Astrakhan, Russia
[6]Institute of Bioorganic Chemistry, Tashkent, Uzbekistan
[7]Center of High Technologies, Tashkent, Uzbekistan
[8]Institute of Humanitarian Research of the Republic of Bashkortostan, Ufa, Russia
[9]Forensic science centre of the Ministry of Justice of the Republic of Kazakhstan, Astana, Republic of Kazakhstan
[10]The Wellcome Trust Sanger Institute, Wellcome Genome Campus, Hinxton, United Kingdom

¶Contributed equally
*Corresponding authors: balanovsky@inbox.ru (OlegB) and mzhabagin@gmail.com (MZ)

Supplementary Text

Historical and ethnographic description of the Kazakh and Turkmen clans studied

by Zhaxylyk Sabitov, Zhabagin Maxat and Oleg Balanovsky

The social structure of the tribal-clan group was the key structural element of political organization in Eurasian steppe civilization. This structure was hierarchical and included tribes consisting of clans, which, in turn, consisted of genealogical lineages. A lineage is a group of people who consider themselves to be descendants of a common ancestor. Most steppe genealogies, historical and ethnographic descriptions of clans or tribes link all descendants to the tribe founder. However, lineage and clan are social, rather than biological, entities. A sequence of ancestors indicated in the oral genealogical tradition might or might not coincide with the genetic (biological) relationships. The patronymic tradition is that clan name is inherited from father to sons - exactly like the Y-chromosome. That is why in cases when a social clan originated from a single biological ancestor, the clan name is a quasi-genetic marker of the ancestor's Y-chromosome.

Here we present historical, ethnographic and genealogical information on the tribes genetically analyzed in this study.

Konyrat tribe

The tribe name reflects an ethno-cultural relationship with the Mongolian tribe Qongirat. This tribe, according to written records, originated from the Darlekin Mongols. Darlekin, in turn, were in a marital alliance with the ruling tribe, the Nyrun Mongols, which included the clan of Genghis Khan [1]. The first wife of Genghis Khan, Borte, was also from the Qongirat tribe (Supplementary Text Fig. 1). Around 1220, Genghis Khan ordered one of Borte's nephews (Tinim-huregene, son of Terkamal) to move from East Asia to the area of Genghis' eldest son Jochi in present-day Kazakhstan [2]. Nanguday [3] - descendant of Tinim in the fourth generation [2] - is known from historical records as emir of the Golden Horde khans, and died in 1359 [4]. Some sources indicate that Nanguday had left 32 sons, some of whom established the Suphy dynasty which ruled in Khorezm up to the 16th century. The Kazakh tribe Konyrat, according to genealogical tradition, originated from two other sons of Nanguday: Zhygaly and Meldebi (Supplementary Text Fig. 1). Zhygaly became ancestor of the Kulshigash clan, and Meldebi became ancestor of two clans: Koktynuly and Kotenshi [3]. Note that the Uzbek Konyrats, who ruled in Khorezm in the 19th century, originated from another of Nanguday' sons - Kharun-myrza [2]. At the turn of the 19th century the census size of the Kazakh Konyrat was around 150,000 [5].

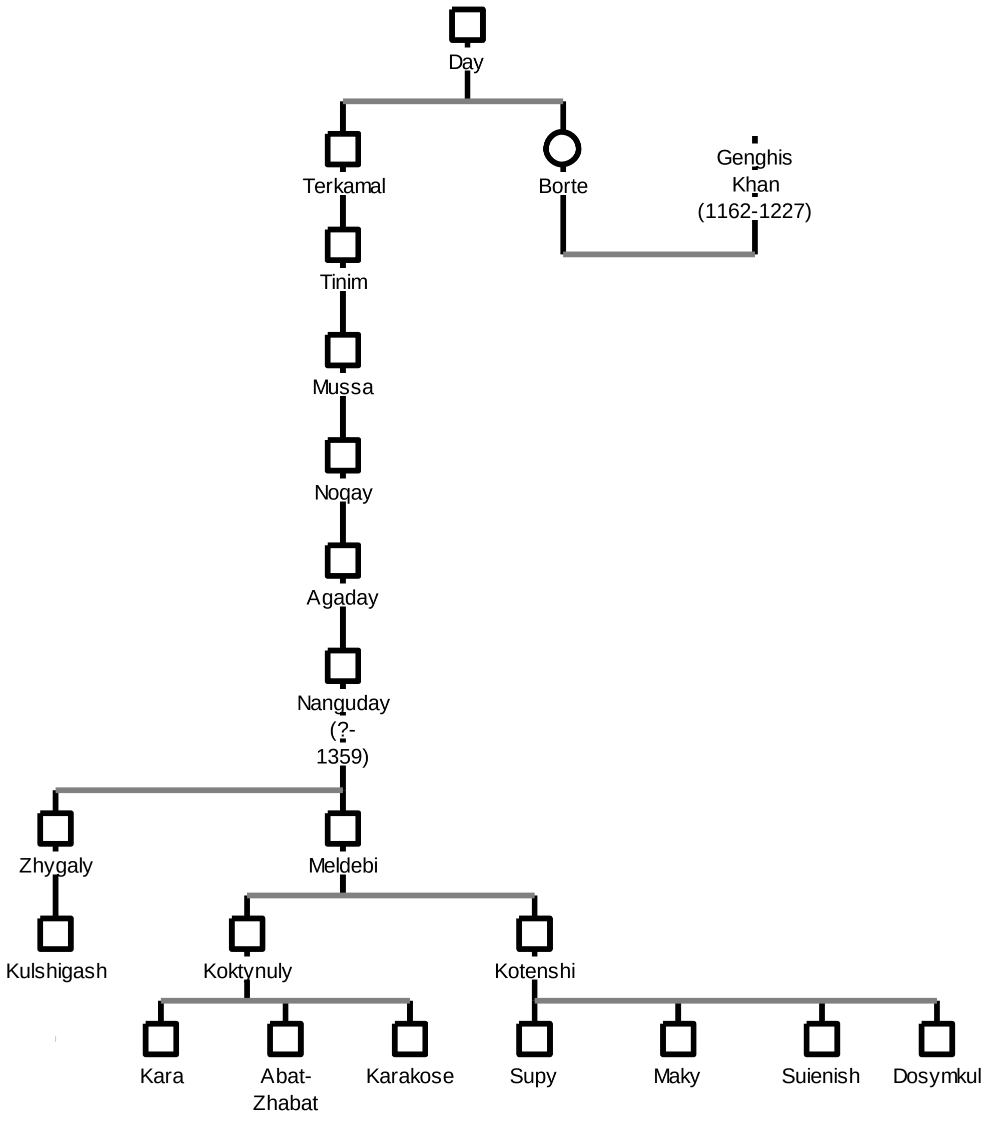

Supplementary Text Figure 1. Genealogical scheme of the Konyrat tribe.

Kozha-Sumak clan group

According to genealogical tradition, the Kazakh clans Kozha and Sunak originated from Near Eastern Muslim missionaries, who belonged to the Quraysh tribe and were paternal-line relatives of the Prophet Muhammad; these Kazakh clans were thus Sayeds (Supplementary Text Fig. 2) [3, 6-8]. Previous genetic studies of Sayeds from Pakistan and India have not revealed a recent common paternal ancestor, but they were genetically closer to Arabs than to the control samples from Pakistani and Indian populations [9]. Our preliminary analysis of the Y-chromosomal gene pool of the Kozha clan revealed the prevalence of haplogroups R1a, J2, and R2a indicating a likely West Asian origin (Middle East, Iran, or Tajikistan); thus missionaries who brought Islam to Transoxiana might have originated from these regions [10].

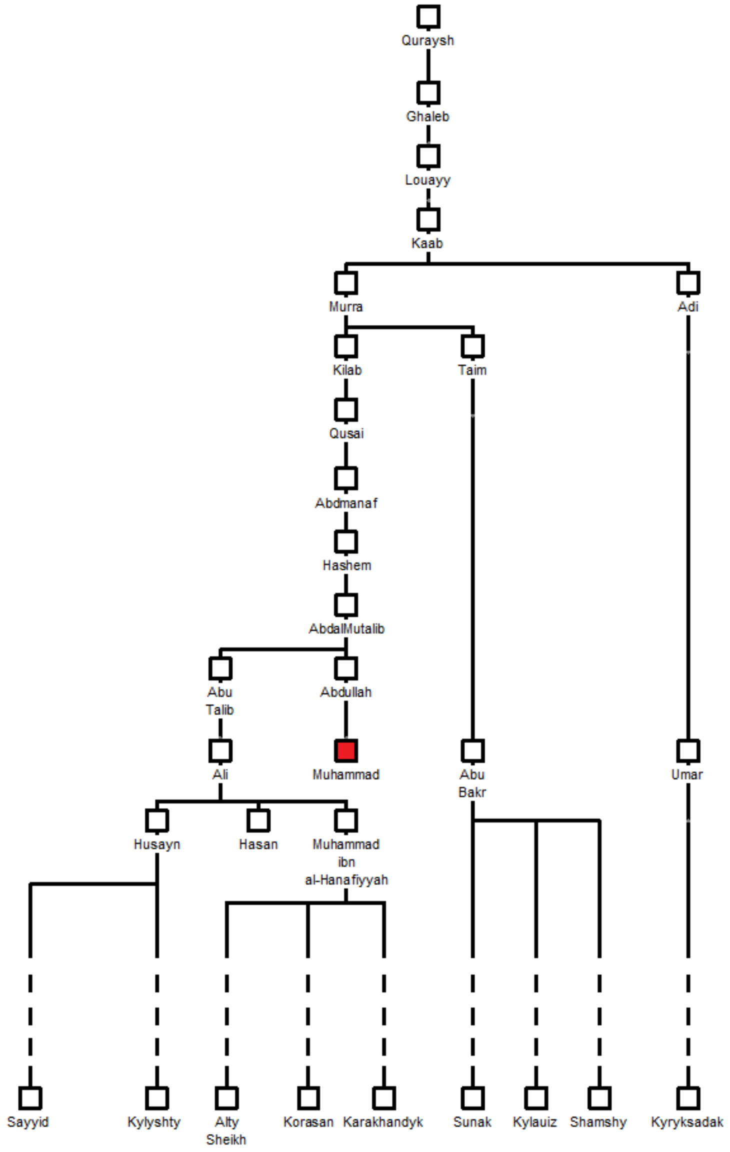

Supplementary Text Figure 2. Genealogical scheme of the Quraysh from the Eurasian steppe. The red square designates Prophet Muhammad's position on the genealogy. Dashed lines indicate dynasties from which lineages of the Kozha and Sunak clans originated.

Alimuly clan

According to genealogical studies, the Alimuly are part of the large Alshin tribe [5], which supposedly migrated to the area of present-day Kazakhstan from East Asia [1] in the 13th century [11]. “Alimuly” can be translated as "children of Alim". Historical records also indicate other names of the Alimuly tribe: "Chumyn" and "Chekly". Genealogical legends record that the ancestor of the Alimuly - Alau – who was emir of Janibek khan (1342-1357), and his son Kyduar (Supplementary Text Fig. 3) was already an old man in 1420 [12]. After the Golden Horde collapsed, Alimuly leaders in the beginning of the 17th century were in social and possibly in biological relationships with the Uzbekistan rulers (for example, the status of the Alimuly leader Jalantos was as high as that of Genghis Khan’s descendants). When Jalantos died around 1660, Alimuly joined the Kazakhs. Nowadays, the Alimuly clan consists of six lineages: Kete, Shomekey, Shekty (also known as "Chekly" [13]), Tortkara, Karakesek, and Karasakal (Supplementary Text Fig. 3).

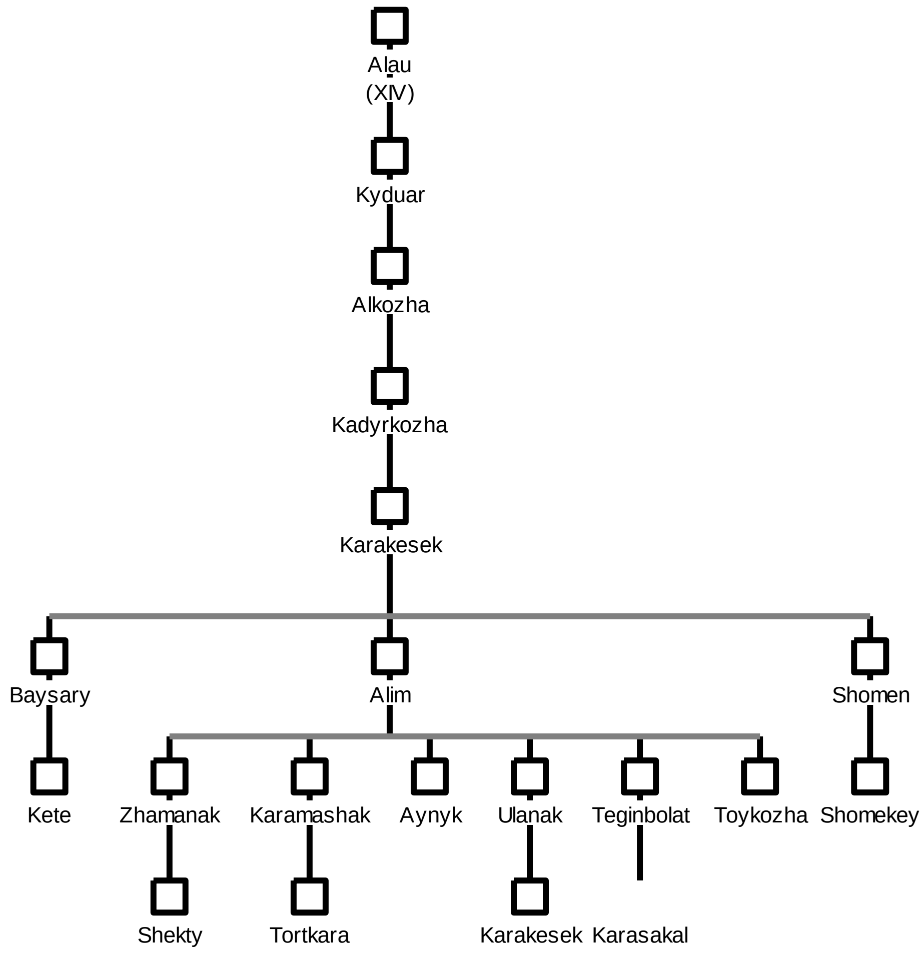

Supplementary Text Figure 3. Genealogical scheme of the Alimuly clan.

Yomut tribe

The Yomut (Yomud) are one of the five large tribes of Turkmens, who dwelt in the Astrabad region (in the area of present-day Iran). Historical evidence indicates that their ancestor Yomut lived at the turn of the 8th century [13]. It is also known that the Oghuz, who were ancestors of the Turkmen, were defeated by the Karluks in the 8th century, and migrated from south-eastern Kazakhstan to lands along the Syr Darya river [14]. In the 13th century, the Yomuts were under the rule of the Golden Horde Batu Khan [15]. By the beginning of the 18th century, the Yomut tribe had split. One part migrated to Khorezm, while another part stayed in southwest Turkmenia [16].

References

1. Rashid al-Din. *Collection of Histories.* (ed. Semenov A. A.) Vol.1, book 1. (Publishing house of the Academy of Sciences of the USSR, 1952).
2. Munis, K. and Agahi, M. *Firdaws Al-Iqbal: History of Khorezm.* (ed. Bregel Y.) (Brill, 1999).
3. Beysenbayuly, Zh. *Qazaq genealogies*. (Ana tili, 1994).
4. *History of Kazakhstan in the Persian sources.* (ed. Kul-Mukhamed M.) Vol.4 (Daik-Press, 2006).
5. Massanov, N. E. *Nomadic Civilization of Kazakhs: the basics migratory habits of life of society.* (Nurbolat Masanov Fund, 2011).
6. Bolshakov, O. G. *The history of the Caliphate: Islam in Arabia (570-633).* Vol.1 (Eastern Literature, 2002).
7. Muminov, A. K. *Family Tree of Mukhtar Auezov*. (ed. Abashin S. N.) (Zhibek Zholy, 2011).
8. Yerofeyeva, I. V. *Genealogies of Kazakh khans and Kozha in XVIII-XIX centuries (history, historiography, sources).* (Print-S, 2003).
9. Belle, E. M. S., Shah, S., Parfitt, T. & Thomas, M. G. Y chromosomes of self-identified Syeds from the Indian subcontinent show evidence of elevated Arab ancestry but not of a recent common patrilineal origin. *Archaeological and Anthropological Sciences* **2**, 217-224, doi:10.1007/s12520-010-0040-1 (2010).
10. Zhabagin, M. et al. The relation between the Y-chromosomal variation and the clan structure: the gene pool of the steppe aristocracy and the steppe clergy of the Kazakhs. *Moscow University Anthropology Bulletin.* **1**, 96-101 (2014)
11. Tynyshpaev, M. *Materials on the history of Kyrgyz-kazakh people.* (Eastern branch of the Kyrgyz State Publishing, 1925).
12. Iskhakov, D. M. and Izmailov, I. L. *Ethno-Political History of the Tartars*. (III- middle of XVI century.) (School, 2007).
13. Sabitov, Zh. M. Alshins (Alchi-Tatars) in the history of the Volga and Ural regions in the XIII-XIX centuries. *Historical destiny of the peoples of the Volga and Ural regions*. **5**, 383-393. (2015)
14. *The history of Kazakhstan from ancient times to the present day in five volumes*. (ed. Kozybayev M.) Vol.1. (Ata-mura, 2010).
15. Dzhikiev, A. *Essays on origin and formation of the Turkmen people in the Middle Ages*. (Gylym, 1991).
16. *The Cambridge History of Inner Asia: The Chinggisid Age.* (ed. Di Cosmo N., Frank A. J., Golden P. B.) (Cambridge University Press. 2015).

Supplementary Figures

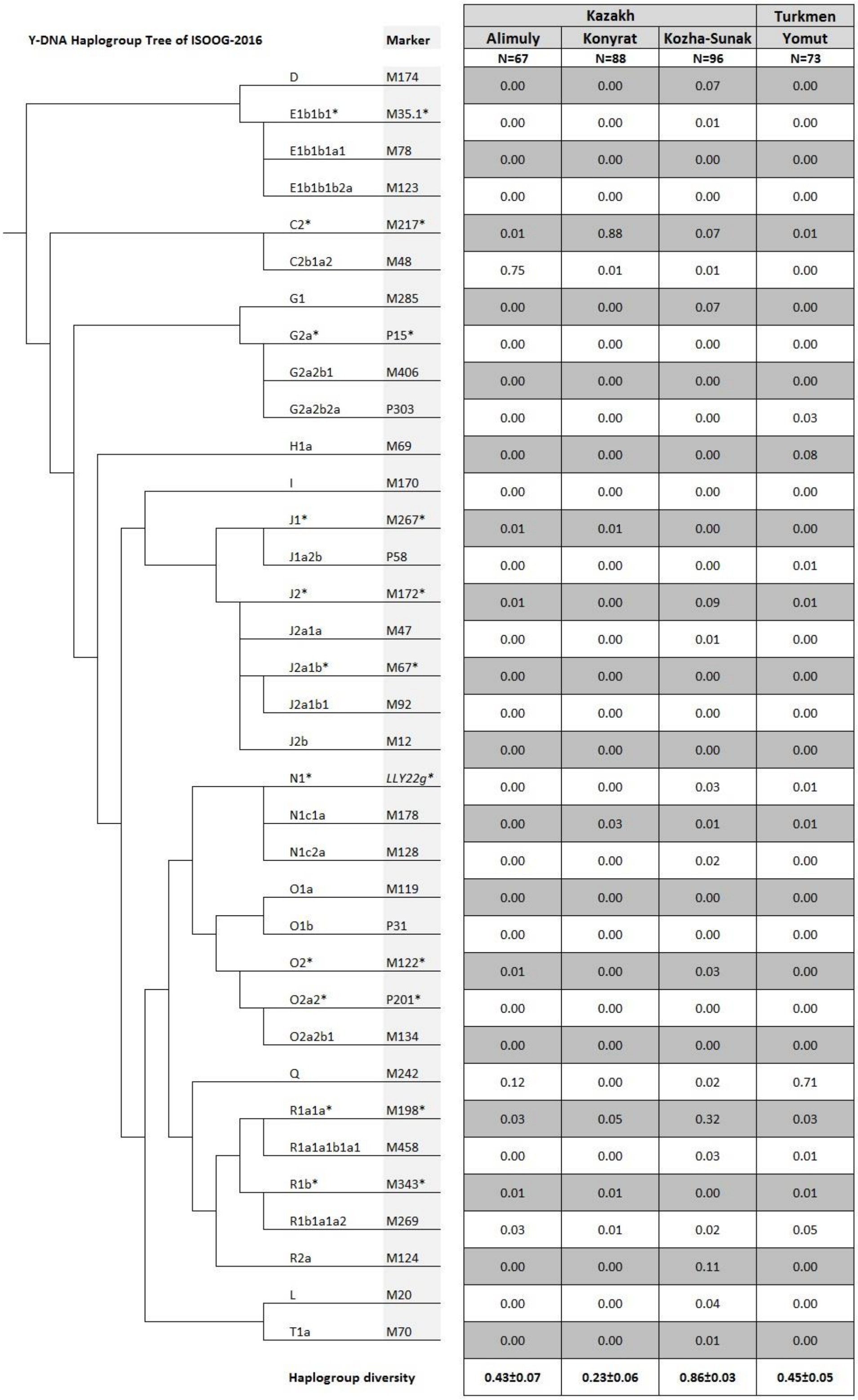

Y-DNA Haplogroup Tree of ISOOG-2016	Marker	Kazakh Alimuly N=67	Kazakh Konyrat N=88	Kazakh Kozha-Sunak N=96	Turkmen Yomut N=73
D	M174	0.00	0.00	0.07	0.00
E1b1b1*	M35.1*	0.00	0.00	0.01	0.00
E1b1b1a1	M78	0.00	0.00	0.00	0.00
E1b1b1b2a	M123	0.00	0.00	0.00	0.00
C2*	M217*	0.01	0.88	0.07	0.01
C2b1a2	M48	0.75	0.01	0.01	0.00
G1	M285	0.00	0.00	0.07	0.00
G2a*	P15*	0.00	0.00	0.00	0.00
G2a2b1	M406	0.00	0.00	0.00	0.00
G2a2b2a	P303	0.00	0.00	0.00	0.03
H1a	M69	0.00	0.00	0.00	0.08
I	M170	0.00	0.00	0.00	0.00
J1*	M267*	0.01	0.01	0.00	0.00
J1a2b	P58	0.00	0.00	0.00	0.01
J2*	M172*	0.01	0.00	0.09	0.01
J2a1a	M47	0.00	0.00	0.01	0.00
J2a1b*	M67*	0.00	0.00	0.00	0.00
J2a1b1	M92	0.00	0.00	0.00	0.00
J2b	M12	0.00	0.00	0.00	0.00
N1*	*LLY22g**	0.00	0.00	0.03	0.01
N1c1a	M178	0.00	0.03	0.01	0.01
N1c2a	M128	0.00	0.00	0.02	0.00
O1a	M119	0.00	0.00	0.00	0.00
O1b	P31	0.00	0.00	0.00	0.00
O2*	M122*	0.01	0.00	0.03	0.00
O2a2*	P201*	0.00	0.00	0.00	0.00
O2a2b1	M134	0.00	0.00	0.00	0.00
Q	M242	0.12	0.00	0.02	0.71
R1a1a*	M198*	0.03	0.05	0.32	0.03
R1a1a1b1a1	M458	0.00	0.00	0.03	0.01
R1b*	M343*	0.01	0.01	0.00	0.01
R1b1a1a2	M269	0.03	0.01	0.02	0.05
R2a	M124	0.00	0.00	0.11	0.00
L	M20	0.00	0.00	0.04	0.00
T1a	M70	0.00	0.00	0.01	0.00
Haplogroup diversity		0.43±0.07	0.23±0.06	0.86±0.03	0.45±0.05

Supplementary Figure 1. Frequencies of Y-chromosomal haplogroups in the tribal/clan groups of Transoxiana.

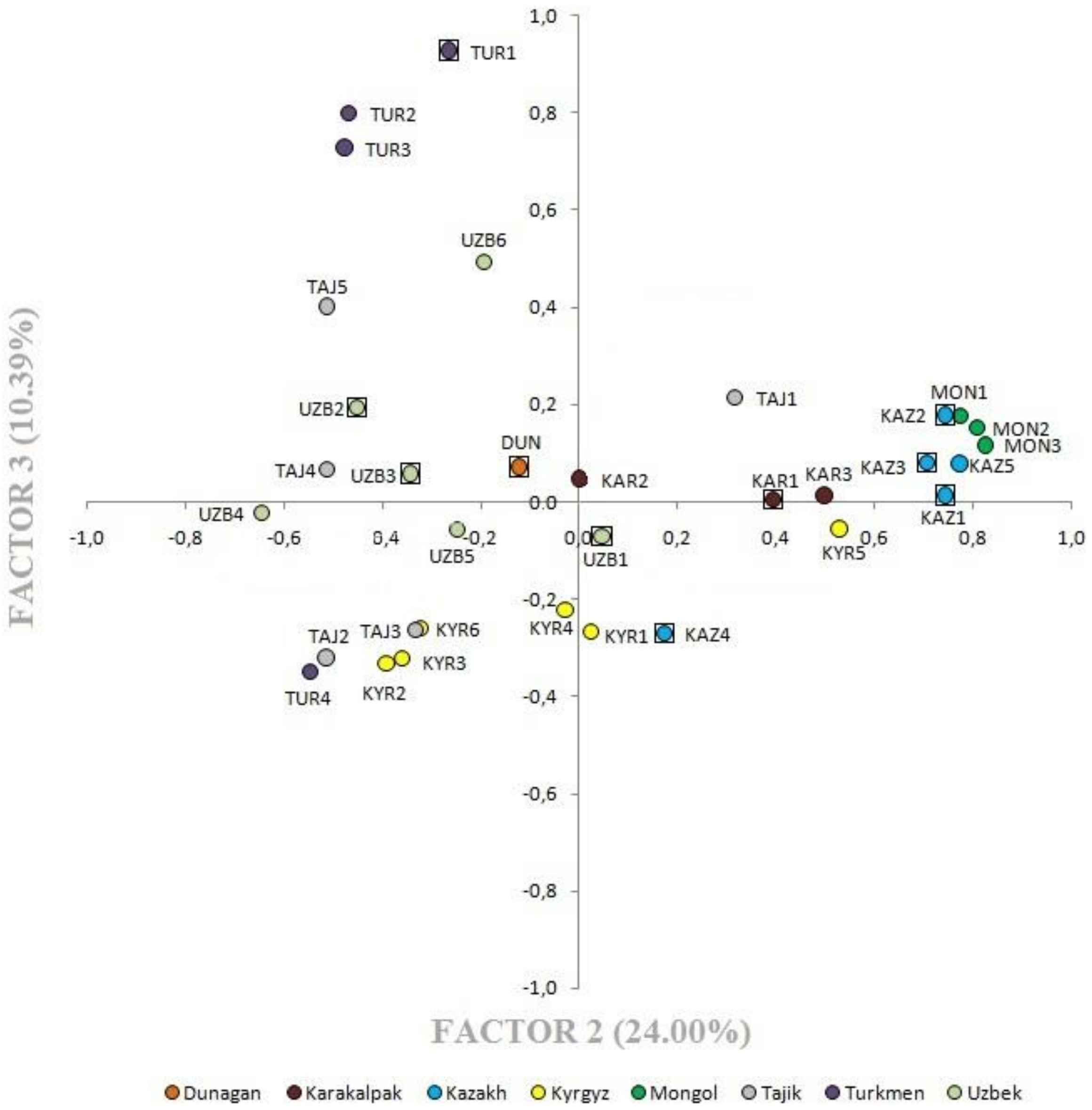

Supplementary Figure 2. Genetic relationships between Transoxianan populations. Principal component analysis: PC2&3.

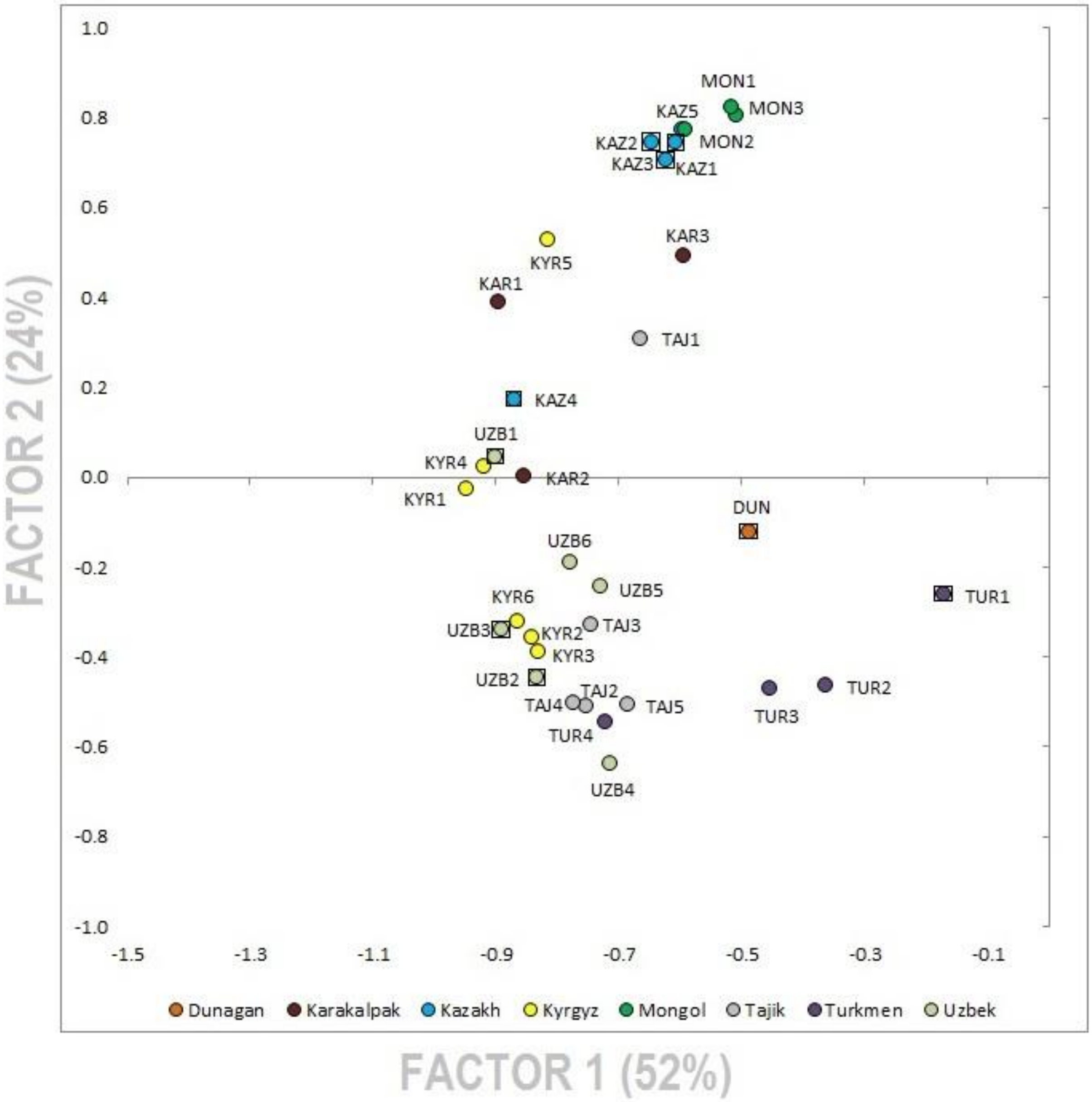

Supplementary Figure 3. Genetic relationships between Transoxianan populations. Principal component analysis: PC1&2.

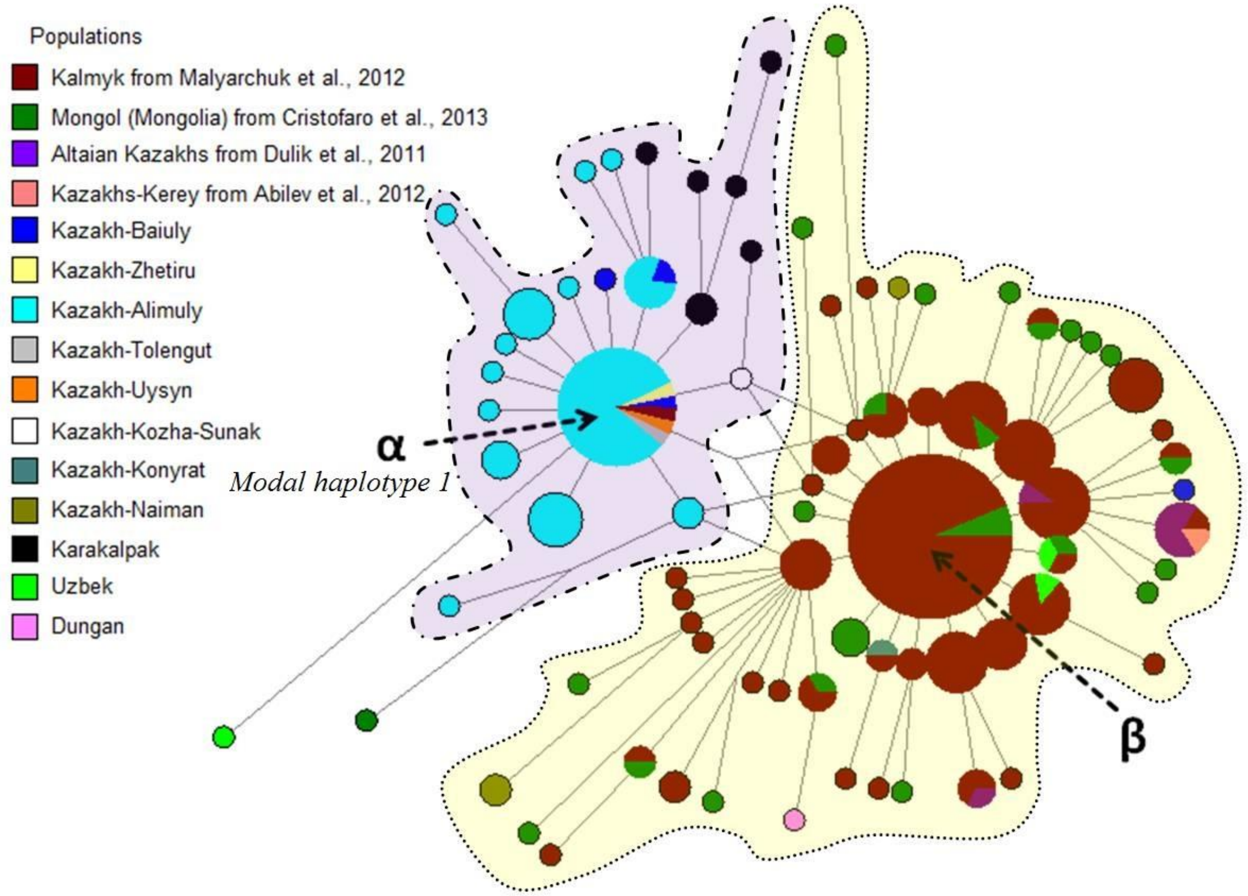

Supplementary Figure 4A. Phylogenetic network of the five-step neighbors of the modal haplotype 1.

Colored contours designate boundaries of the selected clusters, named by the Greek letters. Arrows indicate haplotypes considered as founders for the age calculation using the rho-statistic.

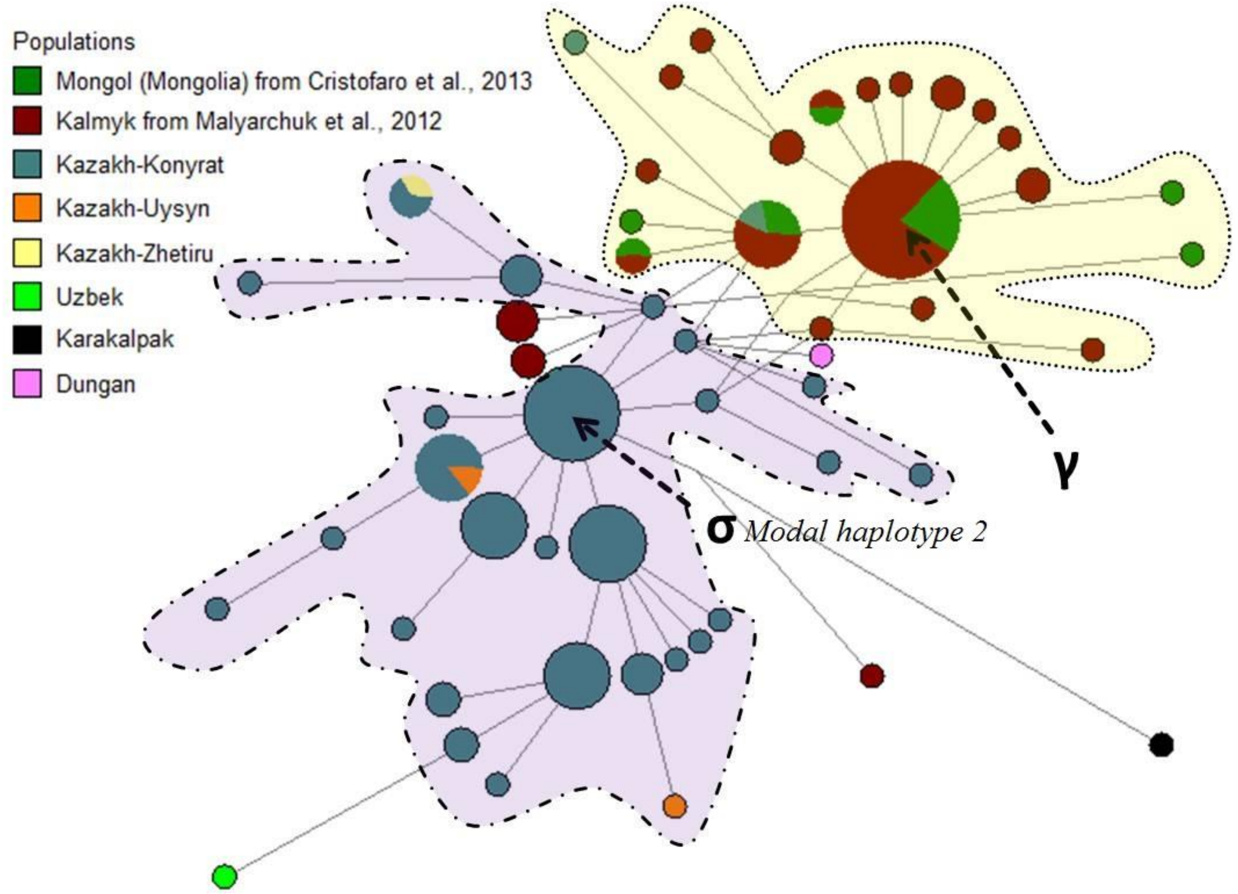

Supplementary Figure 4B. Phylogenetic network of the five-step neighbors of the modal haplotype 2.

Colored contours designate boundaries of the selected clusters, named by the Greek letters. Arrows indicate haplotypes considered as founders for the age calculation using the rho-statistic.

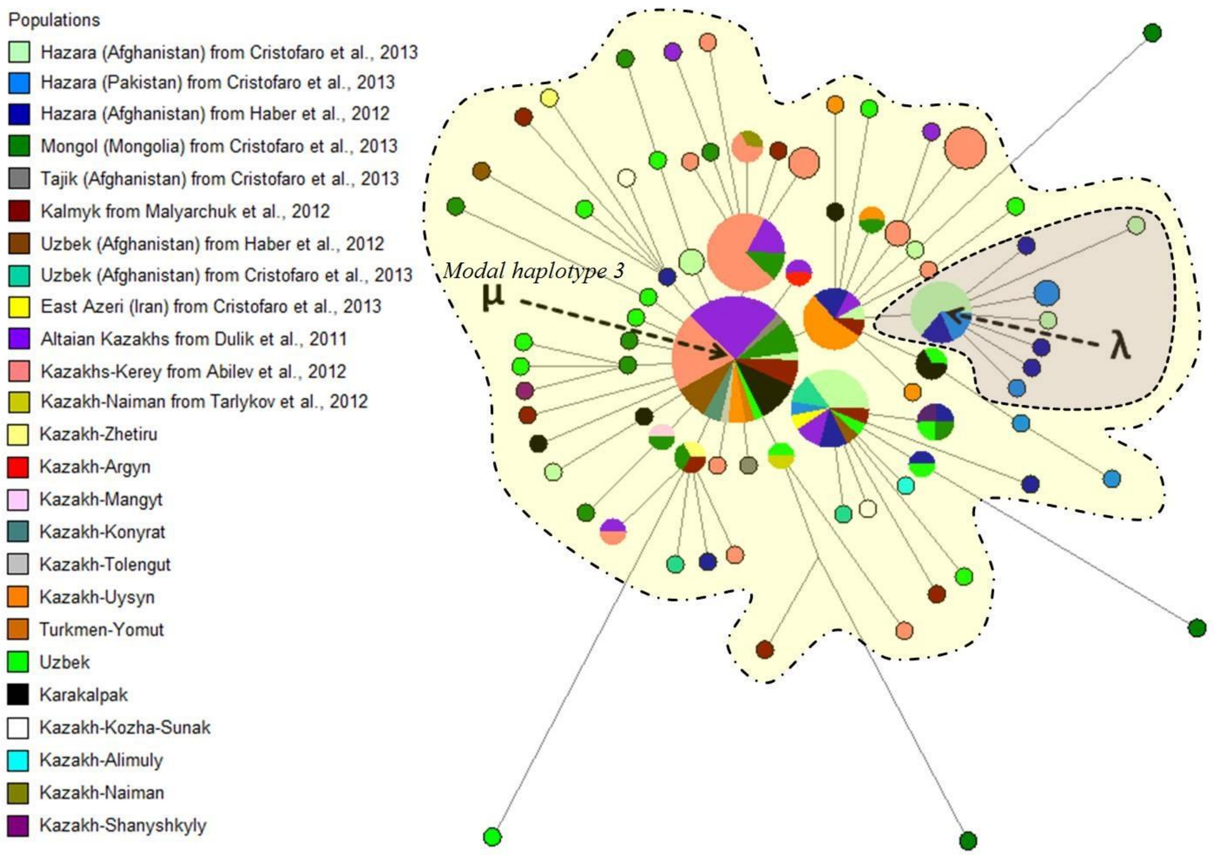

Supplementary Figure 4C. Phylogenetic network of the five-step neighbors of the modal haplotype 3.

Colored contours designate boundaries of the selected clusters, named by the Greek letters. Arrows indicate haplotypes considered as founders for the age calculation using the rho-statistic.

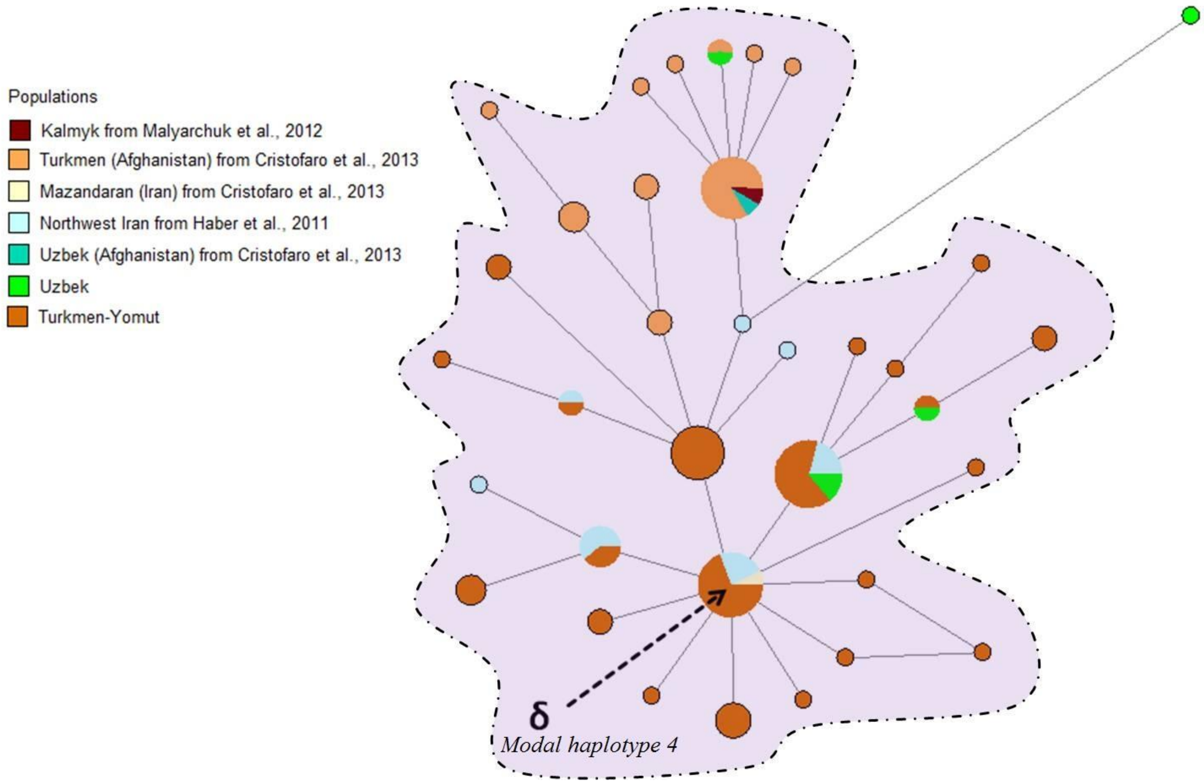

Supplementary Figure 4D. Phylogenetic network of the five-step neighbors of the modal haplotype 4.

Colored contours designate boundaries of the selected clusters, named by the Greek letters. Arrows indicate haplotypes considered as founders for the age calculation using the rho-statistic.
